# Inverse remodelling algorithm identifies habitual manual activities of primates based on metacarpal bone architecture

**DOI:** 10.1007/s10237-018-1091-y

**Published:** 2018-11-09

**Authors:** Alexander Synek, Christopher J. Dunmore, Tracy L. Kivell, Matthew M. Skinner, Dieter H. Pahr

**Affiliations:** 1 Institute of Lightweight Design and Structural Biomechanics, TU Wien, Getreidemarkt 9/BE, Vienna, Austria; 2 Animal Postcranial Evolution Lab, Skeletal Biology Research Centre, School of Anthropology and Conservation, University of Kent, Canterbury, UK; 3 Department of Human Evolution, Max Plank Institute for Evolutionary Anthropology, Leipzig, Germany; 4 Department of Anatomy and Biomechanics, Karl Landsteiner Private University of Health Sciences, Krems an der Donau, Austria

**Keywords:** Micro-finite element, Inverse remodelling, Load estimation, Hand, Metacarpal

## Abstract

Previously, a micro-finite element (micro-FE)-based inverse remodelling method was presented in the literature that reconstructs the loading history of a bone based on its architecture alone. Despite promising preliminary results, it remains unclear whether this method is sensitive enough to detect differences of bone loading related to pathologies or habitual activities. The goal of this study was to test the sensitivity of the inverse remodelling method by predicting joint loading histories of metacarpal bones of species with similar anatomy but clearly distinct habitual hand use. Three groups of habitual hand use were defined using the most representative primate species: manipulation (human), suspensory locomotion (orangutan), and knuckle-walking locomotion (bonobo, chimpanzee, gorilla). Nine to ten micro-computed tomography scans of each species ($n=48$ in total) were used to create micro-FE models of the metacarpal head region. The most probable joint loading history was predicted by optimally scaling six load cases representing joint postures ranging from $-\,75^{\circ }$ (extension) to $+\,75^{\circ }$ (flexion). Predicted mean joint load directions were significantly different between knuckle-walking and non-knuckle-walking groups ($p<0.05$) and in line with expected primary hand postures. Mean joint load magnitudes tended to be larger in species using their hands for locomotion compared to species using them for manipulation. In conclusion, this study shows that the micro-FE-based inverse remodelling method is sensitive enough to detect differences of joint loading related to habitual manual activities of primates and might, therefore, be useful for palaeoanthropologists to reconstruct the behaviour of extinct species and for biomedical applications such as detecting pathological joint loading.

## Introduction

Recently, a micro-finite element (FE)-based inverse remodelling method was developed that reconstructs the loading history of a bone based on its architecture alone (Christen et al 2012; Fischer et al 1998). This method is potentially useful to compute in vivo bone loading required to predict fracture risk (Taddei et al 2014) and fracture healing (Lacroix and Prendergast 2002; Claes et al 1998), or to detect pathological loading conditions (Fischer et al 1999). Since only bone architecture is needed to use the algorithm, it might also be useful for paleoanthropologists to infer knowledge about the behaviour of extinct species where only bone is preserved (Christen et al 2015; Bona et al 2006).

The principle of the inverse remodelling algorithm is based on a simple bone remodelling law (Christen et al 2012, 2014); bone is either added or removed unless the local mechanical stimulus equals a certain remodelling equilibrium stimulus. The goal of the algorithm is, therefore, to find the loading history that most closely leads to remodelling equilibrium within the whole bone. It can be implemented efficiently by computing the load distribution in the bone for a predefined set of load cases using FE models and combining them in an optimal fashion. The method was successfully applied to predict varying in vivo loading conditions in mice vertebrae (Christen et al 2012), was verified on small bone cubes (Christen et al 2013), and delivered reproducible and robust results in distal radius slices (Christen et al 2016). In a recent study, it was also shown that the hip joint loads predicted from whole proximal femora are plausible when compared to in vivo loading measured with instrumented prostheses (Synek and Pahr 2017).

**Fig. 1:**
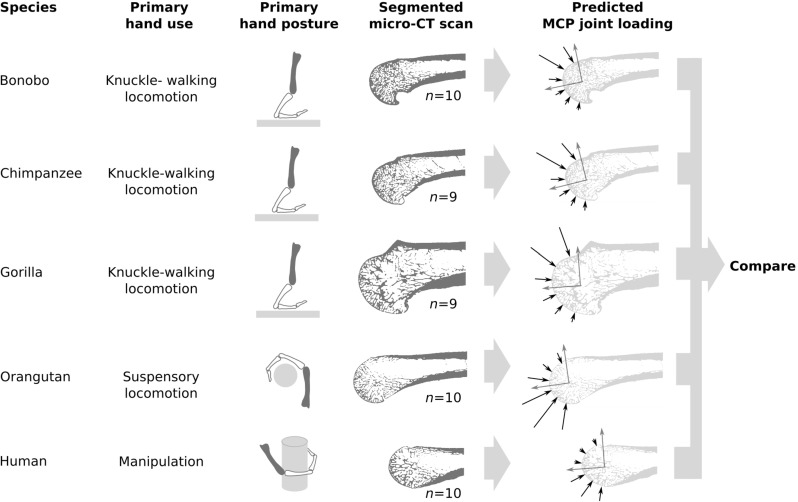
Outline of the study. Metacarpal bones (dark grey in the third column) of five species with different primary hand uses were micro-CT scanned and used to predict the metacarpophalangeal (MCP) joint load history. The black arrows in the rightmost column represent the hypothesized loading history, i.e. predominantly dorsal loading in knuckle-walking species, palmar loading in suspensory and manipulative species, and overall larger loads in species using their hand for locomotion

Although previous studies on the robustness, plausibility, and reproducibility are promising, the accuracy of the algorithm is limited by the number of load cases used. Specifically, using a larger number of load cases was shown to deliver ambiguous rather than more accurate results as the respective load areas start to overlap (Synek and Pahr 2017). Given this limitation, the question remains whether the algorithm is sensitive enough to detect differences of bone loading histories caused by pathologies or different habitual activities.  Christen et al (2016) showed that the predicted loading history well discriminates between bones of either high or low bone volume fraction, but no direct relationship to activity or pathology was drawn. Other studies found qualitative differences in the predicted hip joint loads of varus and valgus patients (Fischer et al 1999) as well as mammalian species with distinct locomotor modes (Bona et al 2006; Christen et al 2015) but were limited to sample sizes as small as a single specimen for each group. As a result, it is still unclear whether the inverse remodelling algorithm is sensitive enough to detect activity- or pathology-related differences in the joint loading history given the coarse nature of the predictions and the lack of variability within the samples tested thus far.

The goal of this study was to fill this gap by applying the micro-FE-based inverse remodelling algorithm to a large sample of bones of various species with broadly similar anatomy, but known differences of habitual activities. More specifically, the loading histories at the metacarpophalangeal (MCP) joints of humans and non-human apes (bonobo, chimpanzee, gorilla, orangutan) were predicted in order to find differences related to primary hand use (manipulation, suspensory locomotion, knuckle-walking locomotion). This joint was chosen due to its anatomical simplicity and previously presented evidence for hand use-related differences of bone architecture (Tsegai et al 2013; Zeininger et al 2011; Chirchir et al 2017; Barak et al 2017). It was hypothesized that: (H1) predicted joint load directions correlate with the expected primary hand postures, and (H2) that predicted joint loads are larger when the hand is used for locomotion when compared to manipulation.

## Materials and methods

### Study outline

The study outline is shown in Fig. 1. Metacarpal bones of five primate species with different primary hand uses were scanned using micro-computed tomography (micro-CT), and the most probable MCP joint loading histories were computed using the micro-FE-based inverse remodelling algorithm originally presented by  Christen et al (2012) and previously adapted and tested for the prediction of joint loads by  Synek and Pahr (2017). The sample was divided into three groups based on the most frequent hand use behaviours: (1) manipulation (humans), (2) suspensory locomotion [orangutans; see  Cant (1987); Thorpe and Crompton (2006)], and (3) knuckle-walking locomotion [bonobos, chimpanzees, gorillas; see  Tuttle (1967); Doran (1996)]. Primarily flexed MCP joint postures were assumed for species using the hand for grasping during manipulation or suspensory locomotion (Napier 1956; Rose 1988) and hyperextended joint postures were assumed for knuckle-walking species (Jenkins and Fleagle 1975) (see Fig. 1, third column). Details about the methodology are presented in the following sections.

### Study sample

Micro-CT scans of nine to ten third metacarpal bones of each species (see Table 1) were obtained using BIR ACTIS 225/300, or Diondo d3 scanners housed in the Department of Human Evolution, Max Planck Institute for Evolutionary Anthropology, Leipzig, Germany, and the Cambridge Biotomography Centre, Cambridge, UK. Specimens were scanned with a voxel size of 24–47 $\upmu$m depending on the size of the specimen. The human sample comprised of four individuals from Nubia Egypt (sixth century to eleventh century), three individuals from Inden, Germany (nineteenth century) and three individuals from Syracuse, Italy (twentieth century). All non-human apes were wild shot, apart from two captive orangutans and one captive bonobo. All specimens included in the study were free of noticeable pathologies.

The sample included both left and right specimens from both sexes as shown in Table 1. Since individual body masses were not available, sex- and species-specific mean values were used in this study (Smith and Jungers 1997). In the two cases where sex was unknown, the average of the male and female body mass was used.

**Table 1:** Overview of the study sample. Five different species were micro-CT scanned and sex- and species-specific average body mass values from  Smith and Jungers (1997) were used

Species		Sample size	Side	Gender	Mean body mass (kg)
Group name	Taxon		(L/R)	(F/M/U)	(F/M/U)
Bonobo	*Pan paniscus*	10	4/6	4/6/0	33.2/45.0/39.1
Chimpanzee	*Pan troglodytes*	9	3/6	5/4/0	40.4/49.6/45.0
Gorilla	*Gorilla gorilla*	9	3/6	5/4/0	80.0/169.4/124.7
Orangutan	*Pongo pygmaeus*, *Pongo abelii*	10	3/7	5/4/1	35.7/78.2/57.0
Human	*Homo sapiens*	10	0/10	2/7/1	54.4/62.2/58.3

The orangutan sample comprised both *Pongo pygmaeus* ($n=8$) and *Pongo abelii* ($n=2$) species

*L/R* left/right, *F/M/U* female/male/unknown

### Image processing

All micro-CT scans were downsampled to 60 $\upmu$m isotropic resolution in Avizo 6.3 (Visualization Sciences Group, SAS) to reduce computational effort without compromising the load prediction results (Christen et al 2016). The scans were filtered with a median filter (support: 2 voxels) and segmented using the Ray Casting Algorithm (Scherf and Tilgner 2009).

**Fig. 2:**
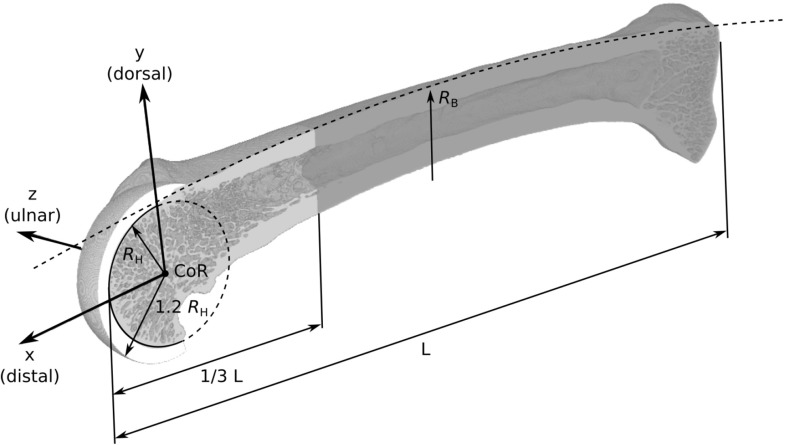
A representative specimen after image processing and defining the MCP joint coordinate system. The MCP joint coordinate system was located at the centre of rotation of the metacarpal head and tilted to account for the dorsal bone curvature (radius $R_\mathrm {B}$)

A custom Python script was then used to find the specimen-specific MCP joint coordinate system in an automated fashion (see Fig. 2). First, the images were further downsampled to 360 $\upmu$m resolution and the voids inside the bone were filled using the fill algorithm of Medtool 4.1 (Dr. Pahr Ingenieurs, Pfaffstätten, Austria). The *x*–*y* plane was computed by finding the plane of the strongest radio-ulnar symmetry of the distal third of the bone using a planar reflective symmetry transform (Podolak et al 2006). The centre of rotation (CoR) and radius of the metacarpal head ($R_\mathrm {H}$) were found by fitting a circle to the distal contour of the bone in the *x*–*y* plane. Finally, the *x*- and *y*-axes of the MCP joint coordinate system were rotated around the *z*-axis to account for intra- and inter-species differences in bone curvature. In particular, a circular arc (radius $R_\mathrm {B}$ in Fig. 2) was fitted to the central part (50% of the bone length *L*) of the dorsal contour of the bone in the *x*–*y* plane. The tilt of the *x*- and *y*-axes was then defined such that the *x*-axis is tangent to the circle fitted to the dorsal contour of the bone.

**Fig. 3:**
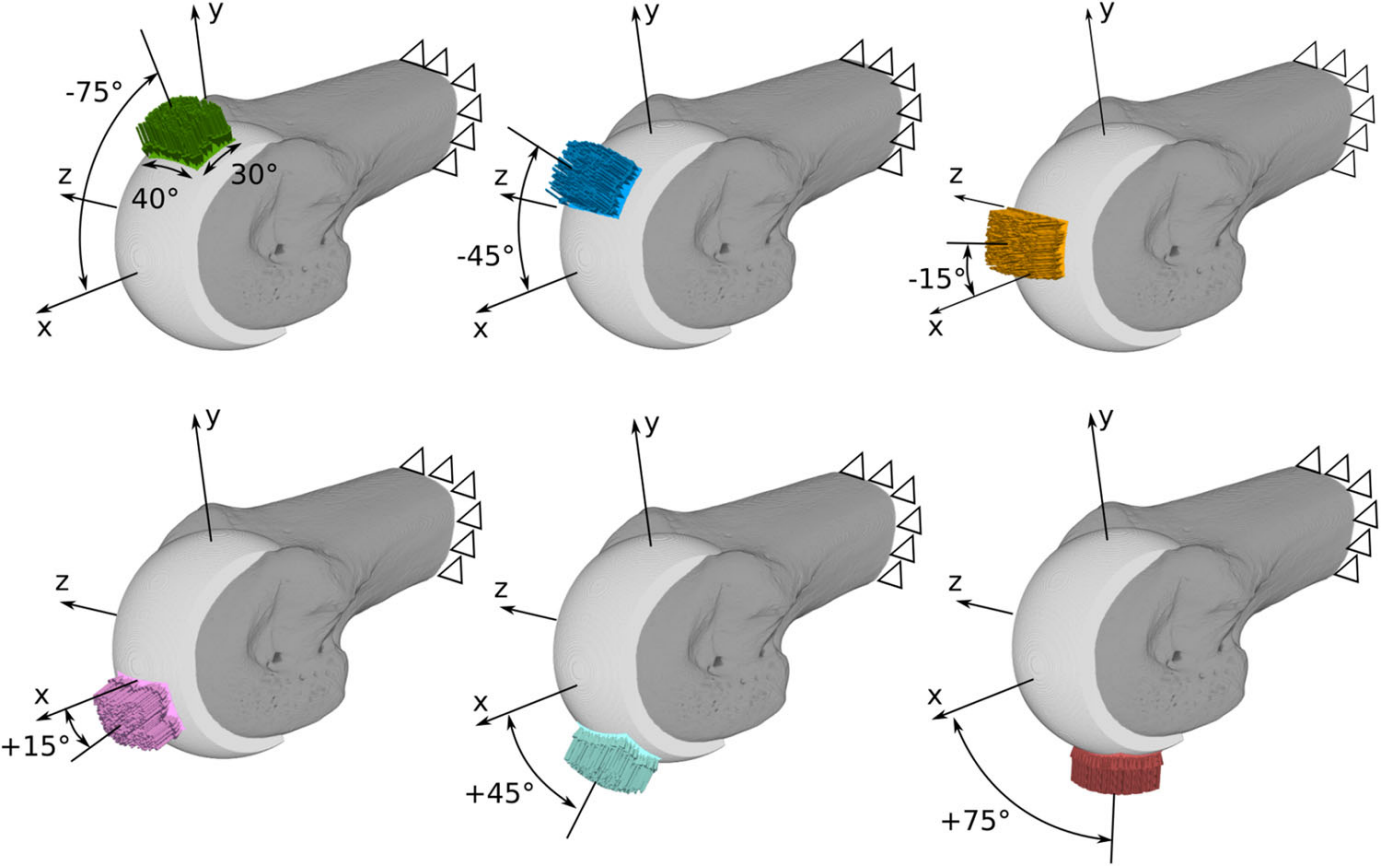
FE models of a single specimen with the six different load cases representing joint loading in postures ranging from highly extended ($-75{}^{\circ }$; top left) to highly flexed ($+75{}^{\circ }$; bottom right)

After definition of the coordinate system, the segmented micro-CT scans (60 $\upmu$m resolution) were cropped to preserve only the distal third of the bone, which contains all or most of the relevant trabecular bone architecture (see Fig. 1, fourth column, and Fig. 2). Finally, a layer of material mimicking cartilage was added to facilitate load application to the FE models. The layer was defined by a sphere located at the CoR of the metacarpal head with a radius of 1.2 times the head radius $R_\mathrm {H}$ (see Fig. 2) and cropped laterally and proximally to remove excess material. The radius of the cartilage sphere was chosen as small as possible but large enough to avoid bone material penetrating through the cartilage surface.

### FE modelling

The processed micro-CT scans were converted into voxel-based micro-FE models with 60 $\upmu$m element side length using Medtool 4.1. Six different load cases were defined for each model, representing joint loading in six postures ranging from $-75^{\circ }$ (extension) to $+75^{\circ }$ (flexion) (see Fig. 3). The proximal end of the bone was fully constrained in all load cases, and forces were applied at the joint surface. All resultant force vectors were within the *x*–*y* plane, pointed to the centre of rotation of the MCP joint, and had a magnitude of 100 N. The force was distributed uniformly on a spherical rectangle ($40^{\circ }\times 30^{\circ }$), and all nodal force vectors were acting in parallel to the resultant force vector. The number of load cases and respective load areas were chosen such that problems associated with overlapping load areas are kept minimal while still providing a reasonable interval and range of load directions to the inverse remodelling algorithm (Synek and Pahr 2017).

The material properties were defined following the previous studies that compared load prediction results with in vivo measurements (Christen et al 2012; Synek and Pahr 2017): the elastic modulus of the bone and the cartilage layer were set to 10 GPa and 10 MPa, respectively, and Poisson’s ratios were set to 0.3 for both materials.

The resulting 288 micro-FE models (48 specimens, six load cases each) with an average of $38.0 \pm 19.7$ million degrees of freedom were solved using the parallel octree solver ParOSol (Flaig 2011). Strain energy densities (SEDs) were evaluated at the element centroids to obtain the load distribution within the bone.

**Fig. 4:**
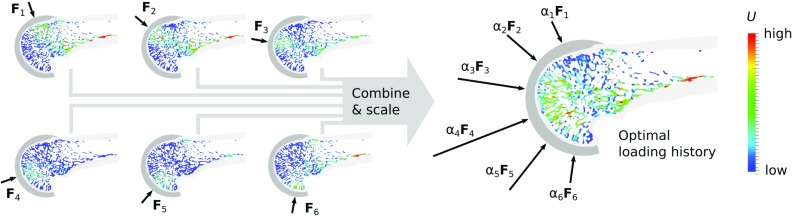
Prediction of the loading history of a single specimen using six load cases representing joint postures ranging from $-75{}^{\circ }$ (extension) to $75{}^{\circ }$ (flexion), with resultant forces $\mathbf {F}_1$ to $\mathbf {F}_6$. The optimal loading history is computed by combining and optimally scaling the load cases such that the distribution of the mechanical stimulus *U* is as homogeneous as possible

### Prediction of the joint loading history

The load history prediction was performed using the inverse remodelling algorithm originally presented by  Christen et al (2012) and previously adapted by  Synek and Pahr (2017). The algorithm is based on the simple remodelling law that bone is either added or removed unless the local mechanical stimulus equals a certain remodelling equilibrium stimulus. Consequently, the most probable bone loading history is the one most closely leading to remodelling equilibrium within the whole bone. A graphical outline of the method is shown in Fig. 4.

The loading history is represented by a finite number of *n* load cases, which are assumed to act with a magnitude $\alpha _i$ for $m_i$ load cycles within an observed time frame. The local mechanical stimulus $U(\mathbf {x})$ at location $\mathbf {x}$ within the bone is computed by summarizing the SEDs $U_i(\mathbf {x})$ resulting from load cases 1 to *n*, weighed by their relative number of load cycles $m_i/m_{\mathrm {tot}}$ and magnitude $\alpha _i$:

1 $$\begin{aligned} U(\mathbf {x}) = \sum \limits _{i=1}^n \frac{m_i}{m_{\mathrm {tot}}} \cdot \alpha ^2_i \cdot U_i(\mathbf {x}) \end{aligned}$$

The most probable loading history for a given bone can then be found by computing the scaling factors $m_i$ and $\alpha _i$ which minimize the difference between the mechanical stimulus $U(\mathbf {x})$ and the remodelling equilibrium stimulus ${\tilde{U}}$ at all locations $\mathbf {x}$ within the bone. This optimization problem can be solved efficiently by introducing the combined scaling factors $s_i = \alpha _i^2 \cdot m_i/m_{\mathrm {tot}}$:

2 $$\begin{aligned} \begin{aligned}&\underset{s_i}{\text {minimize}}&\sum _{\mathbf {x}\in {\mathcal {X}}} \left[ {\tilde{U}} - \left( \sum \limits _{i=1}^n s_i \cdot U_i(\mathbf {x}) \right) \right] ^2 \end{aligned} \end{aligned}$$

Assuming a constant number of load cycles for all *n* load cases (Christen et al 2012; Synek and Pahr 2017), the load magnitude $\alpha _i$ of each load case can then be computed from $s_i$ as follows:

3 $$\begin{aligned} \alpha _i = \sqrt{ n \cdot s_i } \end{aligned}$$

In this study, the remodelling equilibrium stimulus ${\tilde{U}}$ was set to 0.02 MPa as estimated by  Mullender and Huiskes (1995) and used in previous studies (Synek and Pahr 2017; Christen et al 2012). Since the large number of elements in the thick cortex of the diaphysis would introduce a considerable dependency on the model length, only SEDs of trabecular bone elements were considered in the algorithm (see also “Appendix A”). The selection of respective elements was performed using a trabecular bone mask generated using the fill algorithm of Medtool 4.1. The optimization problem presented in Eq. 2 was solved using the non-negative least squares algorithm of SciPy (Jones et al 2001).

The results of the loading history prediction were visualized by scaling the resultant force vector $\mathbf {F}_i$ of each load case *i* with the corresponding load magnitude scaling factor $\alpha _i$ (see Fig. 4). Additionally, a mean joint load vector ${\bar{\mathbf {F}}}$ was computed to compactly represent the loading history and to facilitate inter-specimen comparisons:

4 $$\begin{aligned} {\bar{\mathbf {F}}} = 1/n \cdot \sum \limits _{i=1}^n \alpha _i\mathbf {F}_i \end{aligned}$$

The quality of the load prediction was assessed in terms of the remaining tissue loading inhomogeneity before and after optimizing the load scaling factors. The tissue loading inhomogeneity was quantified by the coefficient of variation (CoV) of the mechanical stimulus *U* (see Eq. 1). A CoV value of 0% would indicate perfectly homogeneous tissue loading (i.e. the whole bone is in a state of remodelling equilibrium).

### Output variables and statistics

Differences in the predicted joint loading histories were assessed both qualitatively and quantitatively in terms of two factors: “hand use” (manipulation, suspensory locomotion, knuckle-walking locomotion) and “species” (human, bonobo, chimpanzee, gorilla, orangutan).

Qualitative comparisons were performed visually using the optimally scaled resultant forces ($\alpha _i \mathbf {F}_i$) for each of the six load cases of each bone. Quantitative comparisons were performed using the mean vector (${\bar{\mathbf {F}}}$) magnitude and direction of each specimen. The mean vector magnitudes were computed both in absolute numbers (i.e. forces) and relative to the species- and sex-specific body mass (i.e. percentage of body weight).

Mean vector magnitudes and directions were statistically compared using one-way ANOVA and Games-Howell post-hoc comparisons in SPSS 23 (IBM Corporation, Somers, NY, USA). The factors “hand use” and “species” were analyzed in separate analyses. The level of significance was set to 0.05.

## Results

### Quality of the joint load predictions

The remaining tissue loading inhomogeneity was successfully reduced in all groups after optimization of the load scaling factors when compared to the initial, uniform load scaling (see Table 2). Despite the reduction, the trabecular bone was still not loaded in a perfectly homogeneous way, with CoV values ranging from 96.7 to 107.5%. However, the remaining tissue loading inhomogeneity after optimization was comparable across species indicating similar quality of the load history prediction.

**Table 2:** Remaining tissue loading inhomogeneity expressed in terms of the coefficient of variation (CoV) before ($\mathrm {CoV_{init}}$) and after optimizing ($\mathrm {CoV_{opt}}$) the load scaling factors

Species	CoV$_\mathrm {init}$ (%)		CoV$_\mathrm {opt}$ (%)	
	Mean	SD	Mean	SD
Bonobo	124.1	13.1	96.7	4.7
Chimpanzee	123.8	12.7	107.5	12.0
Gorilla	111.0	6.5	102.2	4.1
Orangutan	192.5	106.0	104.8	12.1
Human	142.7	39.7	102.9	11.4
Mean	138.8	35.6	102.8	8.8
SD	32.1	41.4	4.0	4.1

*SD* standard deviation

**Fig. 5:**
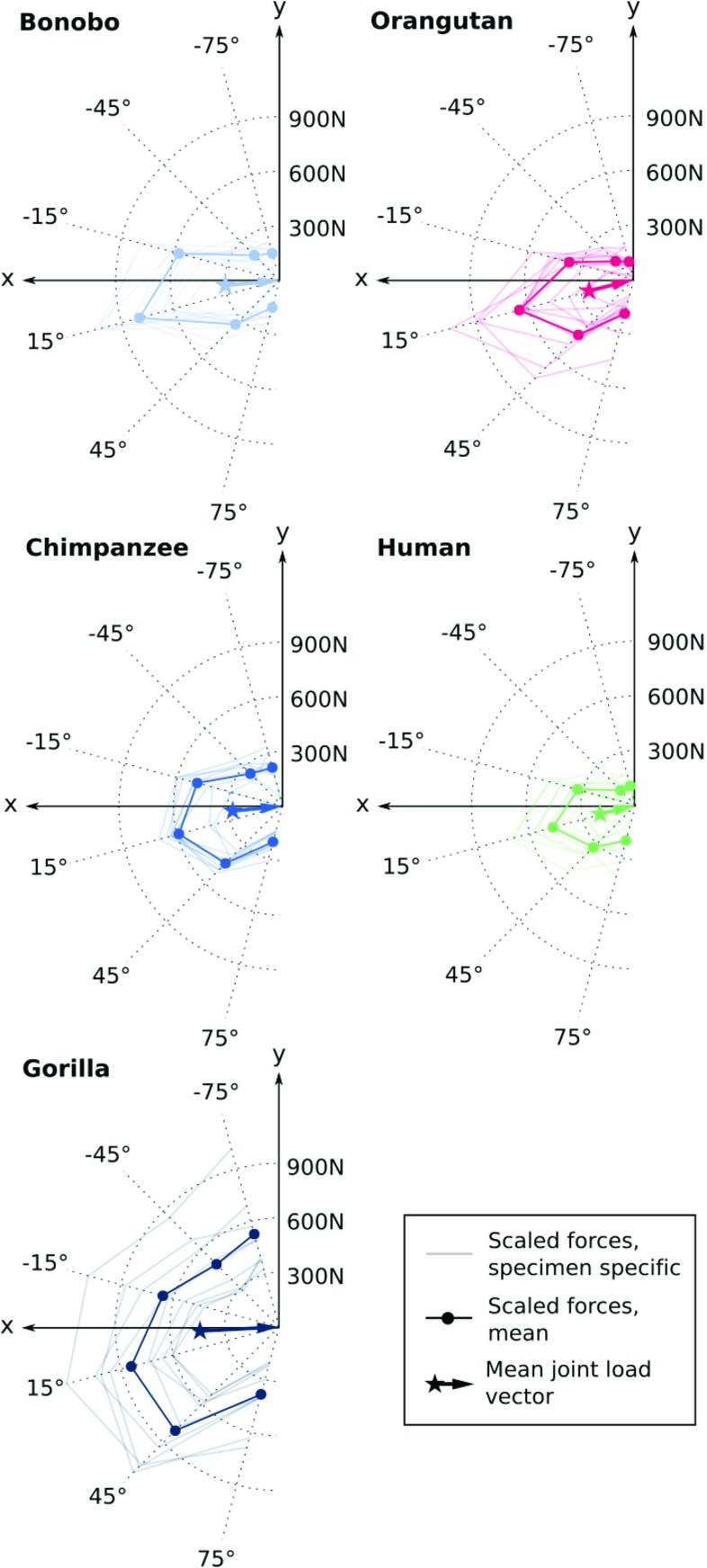
Predicted joint loading histories in terms of optimally scaled resultant forces for each specimen of each species (faint lines) and respective averages (solid lines with filled circles). Additionally, mean joint load vectors of each species are displayed as coloured arrows

**Fig. 6:**
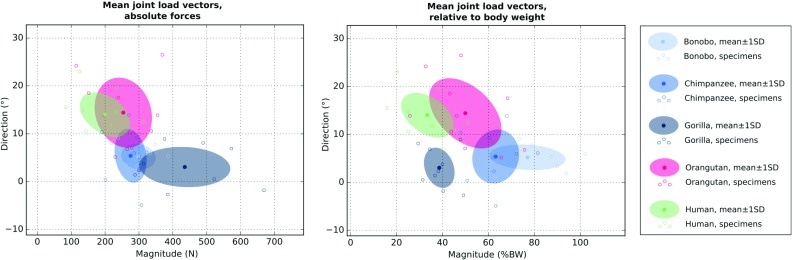
Bivariate plots of the mean joint load vector components (direction, magnitude) for each specimen. Magnitudes are displayed both as forces (left) and relative to body weight (right). Positive and negative direction angles indicate flexion and extension, respectively (see also Fig. 5). Individual species are highlighted by coloured error ellipses scaled to one standard deviation (SD). Shades of blue represent knuckle-walking species (bonobo, chimpanzee, gorilla), pink and green colours represent species using their hand for suspensory locomotion (orangutan) and manipulation (human), respectively

### Qualitative comparison of joint load predictions

The predicted joint loading histories in terms of the optimally scaled resultant forces ($\alpha _i \mathbf {F}_i$) are displayed in Fig. 5. Clear differences between species were observed in the overall load magnitudes, which were largest for the gorillas and smallest for the humans. Other than the load magnitude, the differences in the predicted loading histories were subtle. The peak load was associated with the 15${}^{\circ }$ flexion load case in almost all specimens, and the loading pattern was broadly similar across species. However, slight differences could be observed in terms of the force magnitude ratio of extremely flexed ($+75{}^{\circ }$ load case, factor $\alpha _6$) and extended ($-75{}^{\circ }$ load case, factor $\alpha _1$) postures. In particular, this ratio was larger in species primarily using their hand in flexed postures (human, orangutan; average ratio $\alpha _6/\alpha _1=1.88$) when compared to knuckle-walking species (bonobo, chimpanzee, gorilla; average ratio $\alpha _6/\alpha _1=0.89$).

**Table 3:** *p* Values of all pairwise comparisons of the mean joint load vector magnitudes and directions based on the factors “hand use” and “species”

Factor	Sample 1	Sample 2	Magnitude		Direction
			Abs.	%BW	
Hand use	Knuckle-walking	Manipulation	**0.001**	**0.000**	**0.000**
		Suspension	0.064	0.251	**0.005**
	Manipulation	Suspension	0.306	**0.039**	0.988
Species	Human	Orangutan	0.564	0.098	1.000
		Gorilla	**0.004**	0.251	**0.000**
		Bonobo	**0.023**	**0.000**	**0.001**
		Chimpanzee	0.104	**0.000**	**0.018**
	Orangutan	Gorilla	**0.028**	0.287	**0.006**
		Bonobo	0.620	**0.012**	**0.021**
		Chimpanzee	0.955	0.233	0.052
	Gorilla	Bonobo	0.096	**0.000**	0.680
		Chimpanzee	**0.045**	**0.000**	0.852

Mean joint load vector magnitudes were compared using both the absolute values (scaled forces, labelled “Abs.”) and relative values (percentage of body weight, labelled “%BW”). Significant values ($p<0.05$) are highlighted in bold

### Quantitative comparison of joint load predictions

Quantitative comparisons were performed based on the mean joint load vectors displayed in Fig. 5. To facilitate inter-group comparisons, mean joint load vector directions were plotted against both the absolute and body weight-scaled magnitudes and the groups were highlighted by error ellipses scaled to one standard deviation (see Fig. 6). Despite the large variation within the groups and overall similarity of the predicted loading histories, these bivariate plots demonstrated differences related to primary hand use that will be highlighted in the following.

Knuckle-walking species (bonobo, chimpanzee, gorilla) were characterized by lower mean joint load angles (i.e. more extended MCP joint postures) when compared to species habitually using their hand with a flexed MCP joint for manipulation (human) or suspensory locomotion (orangutan). These differences were significant for the factor “hand use” and all pairwise comparisons of the factor “species” except between the orangutans and chimpanzees (see Table 3).

A tendency towards larger mean joint load magnitudes was observed in species using their hand for locomotion (bonobo, chimpanzee, gorilla, orangutan), particularly if the magnitude was scaled with respect to body weight (see Fig. 6). The latter difference was significant for the factor “hand use” in all pairwise comparisons (see Table 3). However, not all pairwise differences of body weight-scaled load magnitudes were significant for the factor “species”.

## Discussion

The goal of this study was to investigate whether a previously presented micro-FE-based inverse remodelling algorithm is sensitive enough to detect differences of habitual hand use based on the joint loading histories predicted from metacarpal bone architecture. Two hypotheses were investigated for this purpose: first (H1) that the predicted joint load direction would correlate with the primary hand posture and second (H2) that the joint loads would be larger in species using their hand primarily for locomotion compared to those using it for manipulation. Although not as strongly as expected, both hypotheses were supported by this study; mean joint load vector directions were in line with the primary hand postures during knuckle-walking locomotion (more extended MCP joint posture), suspensory locomotion (flexed posture), and manipulation (flexed posture) and mean joint load vector magnitudes tended to be larger in species using their hands for locomotion.

The observed differences in the predicted loading histories are in agreement with previous studies comparing metacarpal bone architectures of various primate species (Tsegai et al 2013; Chirchir et al 2017; Tsegai et al 2017). These studies showed that morphometric differences are small but measurable, particularly with new, holistic approaches to quantify bone architecture (Tsegai et al 2013, 2017). For instance, knuckle-walking species were characterized by overall higher trabecular bone volume fraction and denser subchondral bone in the dorsal regions of the metacarpal head when compared to species using primarily flexed hand postures (Tsegai et al 2013; Chirchir et al 2017). The larger and more dorsally located joint loads predicted for knuckle-walking species in this study are in line with these observations and further support the previously reported sensitivity of the inverse remodelling algorithm on morphometric parameters (Christen et al 2016). While morphometric parameters (e.g. bone volume fraction or degree of anisotropy) alone also allowed discriminating bones of species with distinct hand use to some extent in a recent study (Tsegai et al 2013); the inverse remodelling method has certain advantages which might warrant its application to analyze bone architecture. Firstly, it represents a holistic approach taking into account all features of the bone at once including outer bone geometry, cortical thickness, and trabecular bone structure and thereby eliminates the need for a complex synthesis of the parameters obtained. Secondly, it allows a more direct functional interpretation in terms of both load magnitude and direction even quantitatively without the need to specify multiple regions of interest (Chirchir et al 2017; Barak et al 2017). Particularly the mean joint load vectors might, therefore, be a useful tool to find differences in bone architecture caused by either varying activities or pathologies. Moreover, mean joint load vectors are broadly robust against parameter variations in the inverse remodelling algorithm (Synek and Pahr 2017) and facilitate interpretation of the results as well as inter-specimen and inter-species comparison due to the low number of output variables (e.g. load magnitude and direction). In the present study, these advantages made it possible to find small, but clear differences in the loading histories of species with distinct habitual manual activities.

Although the predicted mean joint load vector magnitudes and directions showed differences related to primary hand use, the extent of these differences was smaller than expected. In particular, the predicted patterns of the loading histories were broadly similar across species and peak values were consistently found for the $+15{}^{\circ }$ load case (see Fig. 5). From a mechanical point of view, it appears reasonable that axial loads are upscaled in the optimization procedure since they cause considerably lower stresses/strains in the bone compared to loads perpendicular to the long bone axis (e.g. compare the SED distribution caused by $\mathbf {F}_4$ and $\mathbf {F}_1$ in Fig. 4). This effect might overrule the comparatively subtle differences of trabecular architecture documented across species (Tsegai et al 2013; Chirchir et al 2017). Another reason for the observed similarities across species might be that the bone architecture is influenced by other manual activities to a larger extent than anticipated. For instance, knuckle-walking is the primary locomotor mode of bonobos, chimpanzees, and gorillas, but all of the species also frequently engage in climbing and suspension as well as object manipulation, in which the hand is using flexed MCP joint postures (Doran 1996; Hunt 1991; Crompton et al 2010). Furthermore, the actual loads acting at the MCP joint during locomotor and manipulative activities are not yet well investigated, particularly in non-human primates. While a correlation between joint load direction and posture appears reasonable due to articular contact, the magnitude of the joint load depends on multiple parameters including external loading, posture, and muscle activity (Chao et al 1989; Weightman and Amis 1982; Qiu and Kamper 2014). Further studies are required to investigate actual differences in joint loads caused by different habitual activities, which will allow a more robust interpretation of the predicted loading histories.

There are several limitations of this study that should be mentioned. Firstly, the load cases used in this study were highly simplified. Actual joint load areas and load distributions are likely more complex and dependent on posture and load magnitude (Tamai et al 1988). Including articular contact in the simulation would potentially lead to more realistic loading conditions (Bona et al 2006), but is considered to be beyond the scope of this study. Instead, an effort was made to standardize the load cases as far as possible to achieve objective inter-species comparisons. Secondly, the inverse remodelling algorithm of  Christen et al (2012) relies on a highly simplified remodelling theory. Although there is evidence that bone formation and resorption are generally related to local mechanical loading (Christen et al 2014; Huiskes et al 2000), the bone architecture is also influenced by other factors such as genetics, calcium homeostasis, and hormone levels (Harada and Rodan 2003; Abel and Macho 2011; Burr 2002; Rodan 1991). Also, the number of load cycles ($m_i$) were assumed to be constant in order to compute the load magnitude scaling factors ($\alpha _i$) following previous publications (Christen et al 2012; Synek and Pahr 2017). This assumption might be interpreted as the dominant influence of the load magnitude on bone formation observed already after a few load cycles (Umemura et al 1997; Rubin and Lanyon 1987), but it remains a limitation of the algorithm owed to the simplified remodelling theory. Additionally, the parameters of the inverse remodelling algorithm were chosen based on previous studies and still require validation. While the choice of parameters has a minor impact on predicted load directions, load magnitudes might be influenced to a larger extent (Synek and Pahr 2017). Reported load magnitudes in this study should, therefore, be considered as a measure of comparison across specimens rather than interpreted in terms of their absolute values. Finally, the study sample was limited to only five species and a single anatomical location. Including comparisons across more species and more anatomical locations (e.g. additional finger joints) could provide further insights into the relation of bone architecture and joint loading histories with respect to habitual activities.

Overall, this study shows that the inverse remodelling algorithm is sensitive enough to detect differences in the joint loading histories caused by distinct habitual manual activities of primates. The method could therefore be particularly useful for palaeoanthropologists to reconstruct behaviour of extinct species, but also for biomedical applications, such as detecting pathological joint loading. However, these applications may constitute additional challenges including the use of poorly preserved bones or low resolution CT scans, which have to be addressed in future studies.

## Figures and Tables

**Fig. 7:**
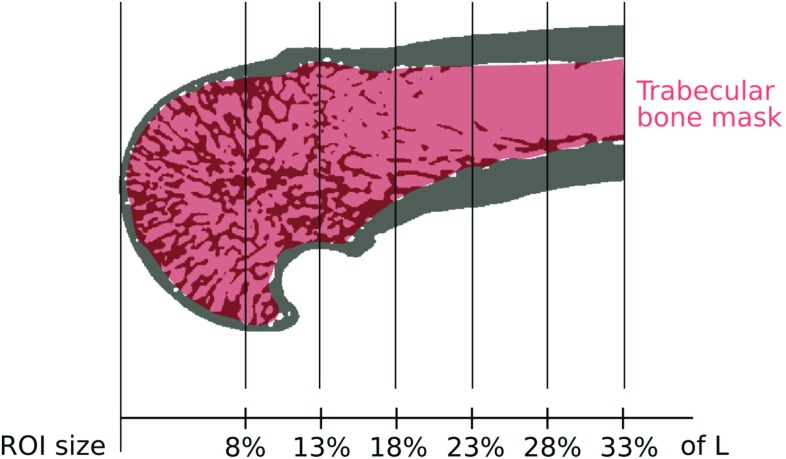
Selection of ROI sizes in this sub-study, displayed on a representative specimen. The red area indicates the trabecular bone region, the variable *L* is the total bone length

**Fig. 8:**
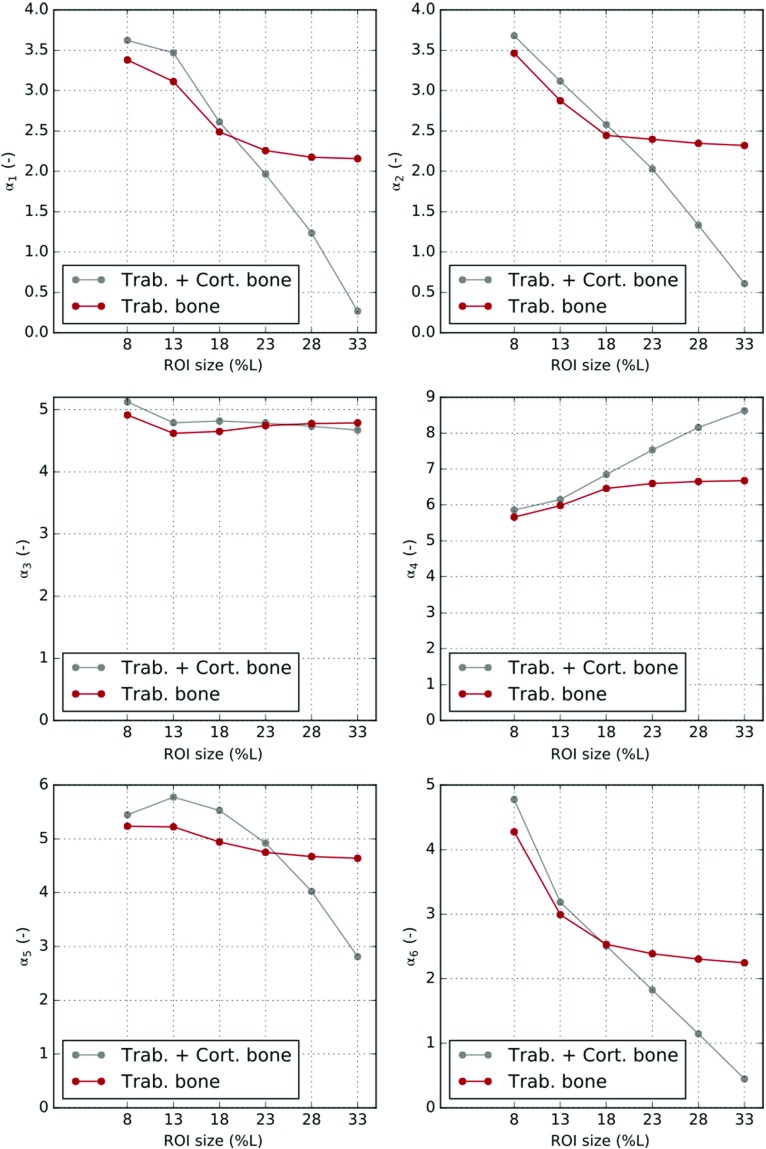
Results of the sub-study investigating the ROI size dependency of the load history prediction. The six plots represent the overall mean ($n=48$) load scaling factors $\alpha _1$ to $\alpha _6$

## Appendix A: ROI size dependency of the load history prediction

In the main text of the manuscript, it is mentioned that only the strain energy densities (SEDs) inside the trabecular bone region were used in the inverse remodelling algorithm. The reason for this choice was to reduce the influence of the selected length of the region of interest (ROI) (see Fig. 7) on the results. The goal of this appendix is to demonstrate the advantage of using only trabecular bone in the algorithm when compared to using both cortical and trabecular bone. For this reason, the load prediction was preformed using the full bone region (cortex and trabecular bone), as well as trabecular bone only for differently sized ROIs. The ROI sizes were reduced from the 33% of the full bone length (as used in the manuscript) to 8% in 5% steps as shown in Fig. 7.

The resulting mean scaling factors $\alpha _1$ to $\alpha _6$ of all 48 specimens are shown in Fig. 8. If the full bone is considered, the ROI size influences the load scaling factors to a large extent. If only trabecular bone is considered, the influence of the ROI size is comparatively small in the range from 18 to 33% of the bone length. Reducing the ROI size further than 18% influences the results obtained both with full and trabecular bone regions. This threshold at an ROI size of roughly 18% well corresponds to the transition from low to high trabecular bone density towards the metacarpal head (see Fig. 7) and highlights the need to include all or most of the trabecular bone in the analysis. Figure 8 also shows that the predicted load scaling factors are quite similar between the full and trabecular bone region for ROI sizes of 18% or smaller.

In conclusion, these results show that using the trabecular bone alone in the algorithm avoids complications related to the selection of the appropriate ROI size while still delivering comparable results to predictions using the full bone region in the metacarpal head area.
